# Effect of introduction of a rapid response system and increasing Medical Emergency Team (MET) activity on mortality over a 20-year period in a paediatric specialist hospital

**DOI:** 10.1016/j.resplu.2024.100823

**Published:** 2024-11-16

**Authors:** Jason Acworth, Connor Ryan, Elliott Acworth, Syeda Farah Zahir

**Affiliations:** a Queensland Children’s Hospital, 501 Stanley St, South Brisbane, QLD 4101, Australia; b Faculty of Medicine, University of Queensland, Herston Rd, Herston, QLD 4006, Australia; c Gold Coast University Hospital, 1 Hospital Blvd, Southport, QLD 4215, Australia; d Centre for Health Services Research, Faculty of Medicine, University of Queensland, QLD, Australia

**Keywords:** Rapid response system, Medical Emergency Team, Paediatric, Clinical deterioration

## Abstract

**Background:**

Rapid Response Systems are hospital-wide patient-focused systems aiming to improve recognition of acute deterioration in patients and trigger a rapid response aimed at preventing potentially avoidable adverse events such as cardiac arrest and death. In 1994, the Royal Children’s Hospital in Brisbane, Australia, was one of the first institutions to adopt a paediatric rapid response system (RRS). The purpose of this study was to investigate the impacts of both introduction of a paediatric RRS and increasing RRS activations (MET dose) on hospital mortality.

**Methods:**

Prospectively collected data from institutional databases at a specialist paediatric hospital was used to determine hospital mortality rate pre- and post- implementation of the RRS. An interrupted time series model using segmented regression was utilised to assess the pre-intervention trend, as well as immediate and sustained effects of RRS implementation on hospital mortality. Univariate linear regression examined potential effects of MET dose on mortality.

**Results:**

Hospital mortality rate did not change significantly over 15 years before RRS implementation. In the first year after implementation, mortality rate fell significantly (−1.4; 95 %CI −2.27 to −0.52; p = 0.0027). For each year that passed after the intervention, there was no significant change in hospital mortality rate (Estimate: −0.08; 95 %CI −0.17 to 0.02; p = 0.11). Univariate linear regression modelling showed that with every unit increase in MET Dose, hospital mortality rate decreased by −0.13 (95 % CI: −0.27 to 0; p = 0.05).

**Conclusions:**

Utilising data from one of the earliest and longest duration single-centre cohort of paediatric MET events, this study reaffirms the association between implementation of a paediatric RRS and decreased hospital mortality. The study also provides novel evidence of the impact of MET dose on patient outcome in the paediatric population. It is recommended that factors influencing the benefit of rapid response systems in paediatric populations are further identified so that this life saving initiative can be optimised.

## Introduction

The Medical Emergency Team (MET) model was first described in Sydney, Australia in the early 1990s and has since been widely implemented around the world.^1^ The concept describes a hospital-wide patient-focused system aiming to improve recognition of acute deterioration in patients and trigger a rapid response from a designated team with critical care skills who enact management aimed at preventing potentially avoidable adverse events such as cardiac arrest and death.^2^

These rapid response systems are different from previous standard “code” or “arrest” team models as they aim to assess patients at an earlier stage of clinical deterioration,^3^ that is, patients with clinical signs of impending respiratory, cardiac or neurological failure, rather than patients who have already suffered a respiratory or cardiac arrest. The model^4^ is particularly suited to paediatric patients because cardiac arrest in this population is more often preceded by a more prolonged period of potentially reversible deterioration and because outcome from cardiac arrest is worse than in adults.^5^, ^6^

In July 1994, the Royal Children’s Hospital (RCH) in Brisbane, Australia replaced its “Cardiac Arrest Team” with a Medical Emergency Team (MET) system. Criteria for activation of the system remained unchanged across the 20 years the system was in place at RCH Brisbane, except for the addition of “CEWT prompt” after a multi-trigger Children’s Early Warning Tool (CEWT) was introduced into the hospital in late 2011. Data collected from all MET responses was prospectively entered into a quality assurance database from February 1995 until July 2014.

The 2020 observational study from the RCH Brisbane MET registry^7^ analysed 771 MET events over 20 years. It identified that patient demographics, reasons for MET activations and procedures performed changed over time but were consistent with the multicentre paediatric MET cohort study from the United States in 2016, reporting on an American Heart Association (AHA) registry.^8^ Across the two decades after implementation of the MET system at the RCH Brisbane, MET events increased significantly and more METs were triggered for patients earlier in their deterioration.^7^

Data from studies describing the implementation of MET systems, now more commonly referred to as Rapid Response Systems (RRS), in paediatric settings has demonstrated the potential positive effects that these early recognition and action systems can have including lower frequency of respiratory and cardiac arrests outside of the PICU, fewer unexpected ward deaths and decreased in-hospital mortality.^9^, ^10^, ^11^, ^12^, ^13^, ^14^

In adult settings, MET dose (defined as MET events per 1000 hospital separations) has been associated with reduced hospital mortality and out of ICU arrest,^15^, ^16^, ^17^, ^18^ but this has not been examined in paediatric populations.

The mechanism by which MET implementation and MET dose achieves improvements in patient-centred outcomes is not yet understood.^19^ As one of the first institutions to adopt a paediatric rapid response system, the dataset from the RCH Brisbane provides a unique opportunity to explore the impacts that introduction of these systems can make on improving patient outcomes. The purpose of this study was to investigate the impacts of both the introduction of a paediatric rapid response system and increasing MET dose on hospital mortality.

## Material and methods

### Source of data

This registry-based study is a single-centre observational study of prospectively collected data entered into an institutional database at the Royal Children’s Hospital in Brisbane, Australia.

The RCH Brisbane MET database collected data related to MET events from February 1995 to July 2014. A previous study utilising this database describes the changes in patient demographics, patterns of activation and clinical outcomes of MET activations.^7^

Data related to annual patient separations (discharges) and hospital mortality from 1980 to 2014 was obtained via the Hospital Based Corporate Information System (HBCIS) from the Queensland Hospital Admitted Patient Data Collection (QHAPDC).

### Inclusion and exclusion criteria

A MET event was defined as any event within the facility for which the MET was activated. The MET response may have been triggered by abnormalities in patient physiology, a subjective concern on the part of the staff, or family/visitor concerns as defined by the hospital’s activation policies or procedures for MET activation.

All MET events for inpatients recorded in the RCH Brisbane MET database from February 1995 to July 2014 were considered eligible for inclusion in the study. Data related to MET events involving paediatric hospital outpatient areas (including the emergency department) as well as MET events to visitors (adults and children) and staff members were excluded. False alarms and duplicate records from the MET database were also excluded from analysis. Additionally, where an interruption or change in data collection had resulted in greater that one month of data loss from the database in any calendar year, that year of data was excluded from analyses related to MET activation frequency (MET dose).

### Data collection and analysis

To determine hospital mortality rate pre- and post- implementation of the RCH MET system, hospital discharge and hospital death data was collected from 1980 (15 years prior to implementation) until November 2014 (20 years after implementation) when the hospital closed. MET activation data was available from February 1995 until July 2014. The RCH MET system (the intervention) was introduced in July 1994, however the intervention was considered fully functional at the start of 1995. To establish an underlying trend, which is ‘interrupted’ by an intervention at a known point in time we used an interrupted time series analysis approach.

The primary outcome, hospital mortality rate, was defined as hospital deaths per 1000 hospital discharges in a given time period (defined as years). The time elapsed since the start of the study was measured in years. An interrupted time series model using segmented regression was used to assess the i) the pre-intervention trend, ii) the level of change following intervention (immediate effect) and iii) the slope following intervention (sustained effect).

A counterfactual model was used to help describe the potential impacts of confounding variables. The counterfactual method provides a hypothetrical estimate of what would have happened without the intervention.^20^ Counterfactuals were built at 10- and 20-years post-intervention to provide an estimate of the hospital mortality had it continued along its previous trajectory, if the RCH MET system had not been implemented.

The association between MET dose and hospital mortality rate was examined using data from 1995 to 2013 (excluding years 2001 and 2014 due to incomplete MET dose data). Initially, correlation (Spearman’s Rank Order Correlation) was conducted to test the strength of association between MET dose and hospital mortality rate. Univariate linear regression was then used to examine the potential effects of MET Dose and hospital mortality rate.

### Ethical approval

The retrospective data collection included only data that was routinely collected as part of a patient’s admission and/or event of a 10.13039/501100015506MET activation. No additional patient data was collected as part of this study. Data extracted from the databases for this study did not include any identifiable data fields. A separate unique study ID number was allocated to each record for this study. The study was approved by the Children’s Health Queensland Human Research Ethics Committee as a Low and Negligible Risk category project [HREC/24/QCHQ/108284].

## Results

### Population characteristics

Between February 1995 and July 2014, a total of 955 MET event records were entered into the registry.^7^ After applying exclusion criteria (112 False Alarms, 9 Duplicate Records), 834 MET events were available for analysis. The median age of patients recorded in this database was 36 months (IQR 10.50–210).^7^

After excluding MET activations to outpatients, emergency patients, staff and visitors, 564 inpatient MET activations were available for calculating annual MET dose for 1995 to 2013 (excluding 2001).

**Fig. 1:**
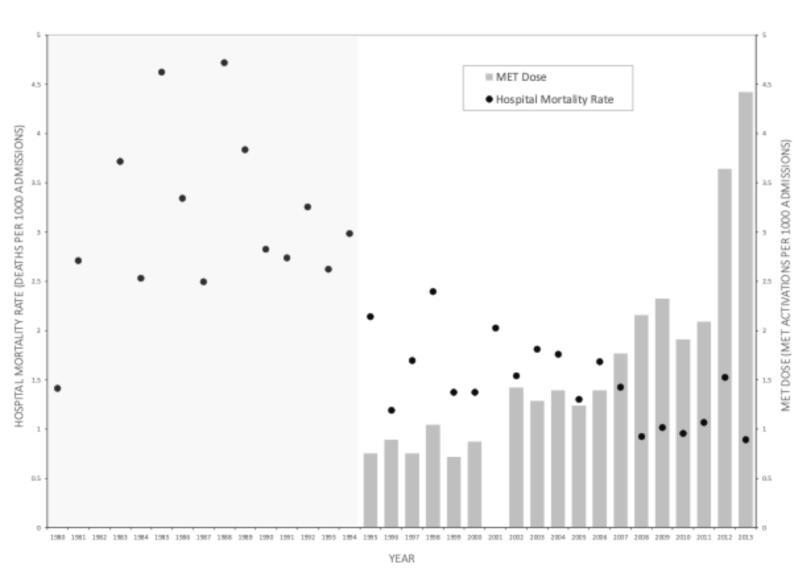
Hospital mortality and MET dose pre- (1980–1994) and post- (1995–2013) MET implementation.

In-hospital cardiac arrests, hospital mortality and MET dose pre- (1980–1994) and post- (1995–2013) MET implementation are shown for each year in Table 1. Hospital mortality rate (1980–2013) and MET dose for each year (1995–2013) after MET implementation is illustrated graphically in Fig. 1.

**Table 1:** In-hospital cardiac arrests, hospital mortality and MET dose pre- (1980–1994) and post- (1995–2013) MET implementation.

1. IHCA rate = in-hospital cardiac arrests per 1000 hospital discharges.

2. IHCA rate external to Crit Care = in-hospital cardiac arrests occurring outside of critical care areas (intensive care, emergency department, operating theatres) per 1000 hospital discharges.

3. Hospital Mortality rate = total deaths per 1000 hospital discharges.

4. MET dose = number of MET events to hospital inpatients per 1000 hospital discharges.

NA Data not available.

### Hospital mortality trends before and after MET implementation

**Fig. 2:**
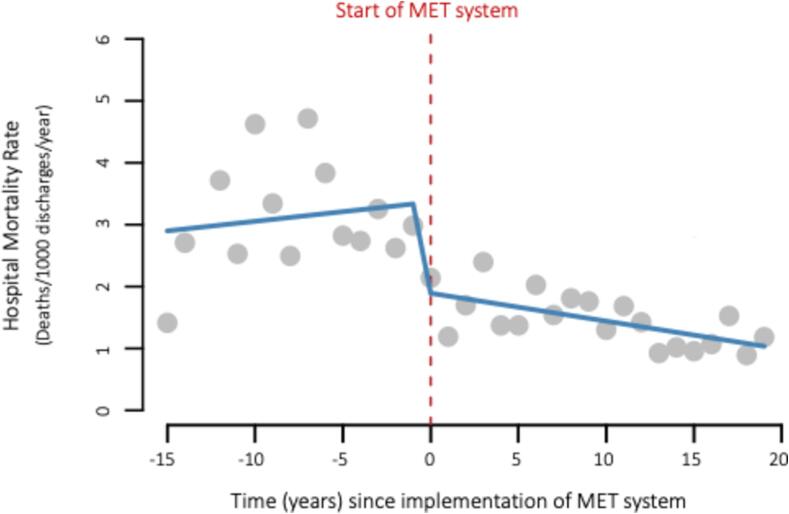
Trends in hospital mortality rate before and after MET implementation.

Hospital mortality rate before and after MET implementation is displayed in Fig. 2.

*Pre-intervention trend:* The hospital mortality rate trend across the 15 years prior to MET implementation was positive and insignificant, indicating that hospital mortality rate did not change significantly over time before intervention (Estimate of change in mortality rate per year: 0.03; 95 %CI −0.05 to 0.11; p = 0.44).

*Immediate effect:* In the first year after implementation of the MET system, the hospital mortality rate fell significantly (Estimate of change in mortality rate: −1.4; 95 %CI −2.27 to −0.52; p = 0.0027).

*Sustained policy effect:* The time since intervention coefficient (the difference between the slope of the line before and the slope of the line after the intervention) showed that for each year that passed after the intervention, there was no significant change in the hospital mortality rate (Estimate: −0.08; 95 %CI −0.17 to 0.02; p = 0.11).

**Fig. 3:**
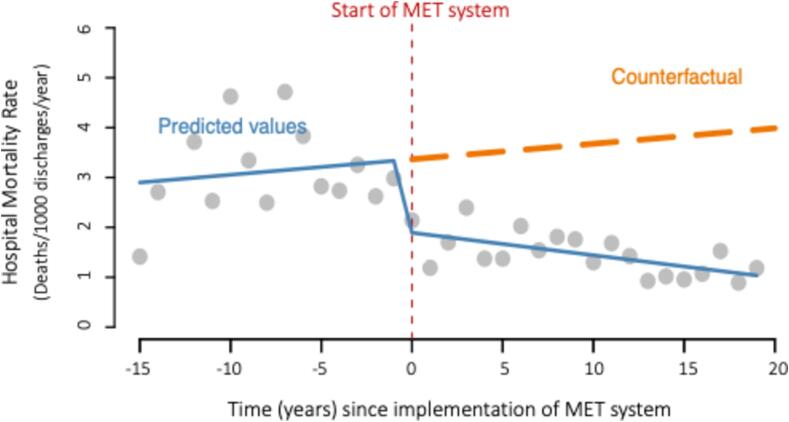
Counterfactual model illustrating hypothetical hospital mortality rate (without MET implementation).

The counterfactual model (showing predicted hospital mortality rate had the MET system not been implemented) is displayed in Fig. 3. The model shows a decreasing mortality rate after the intervention, but the counterfactual (dashed orange line) shows a slight upward continuing trend in hospital mortality (had the MET system not been implemented).

### MET dose and hospital mortality rate

**Fig. 4:**
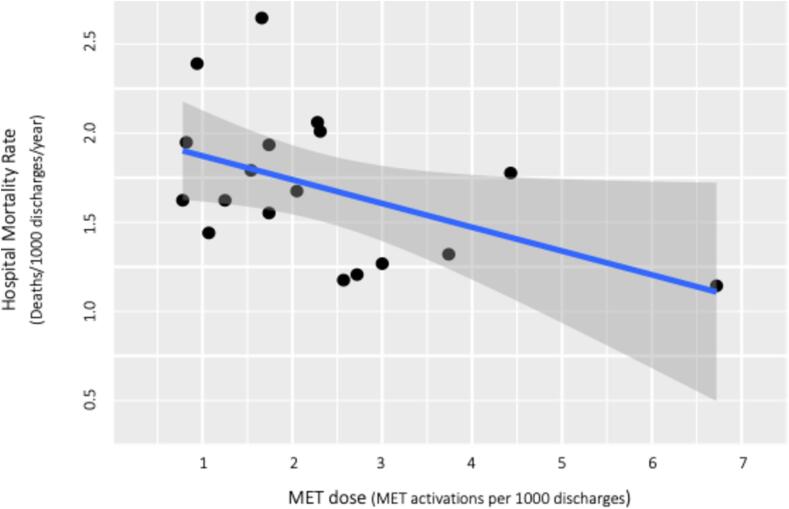
Relationship between hospital mortality rate and MET dose.

The correlation between MET dose and hospital mortality rate (after MET implementation) is displayed in a scatter plot in Fig. 4.

There was a statistically significant moderate negative correlation (Spearman’s correlation) between hospital mortality rate and MET Dose (rho = -0.49, p = 0.04).

The univariate linear regression model used to examine the potential effects of MET Dose on mortality rate indicated that with every unit increase in MET Dose, hospital mortality rate decreased by −0.13 (95 % CI: −0.27 to 0; p = 0.05). The model suggested that 22 % of the variability in hospitality mortality rate could be explained by MET dose (R-squared = 0.22).

## Discussion

This report from the longest duration single-centre cohort of paediatric MET events published to date clearly demonstrated an association between the implementation of a paediatric MET system and a decrease in hospital mortality rate. It also showed a statistically significant correlation between increasing MET dose and decreasing hospital mortality rate. Hospital mortality rate had been steady for 15 years before introduction of the MET system. It then dropped by 40 % within a year of MET introduction, halved by the end of the first 5 years, and was down to about one third of the initial rate by the end of 20 years. This builds upon the existing literature supporting the implementation of MET systems in paediatric hospitals^11-14^ and demonstrates the relevance of MET dose, which was previously unexamined in paediatric populations.

The significant decrease in hospital mortality after introduction of the MET system aligns with findings from other single-centre retrospective cohort studies. A MET service was introduced into the Royal Children’s Hospital (RCH) in Melbourne, Australia in 2002 and reported a 35 % reduction in total hospital mortality and a 65 % reduction in unexpected ward deaths from the 41 months “pre-MET” to the 48 months “post-MET”.^11^

Sharek et al^12^ reported an 18 % decrease in hospital-wide mortality after implementation of a Rapid Response Team (RRT) model. Similarly, Kolovos et al^13^ found a 22 % decrease in PICU mortality following implementation of an RRT model.

A 2010 *meta*-analysis found that use of a paediatric MET system was associated with a 21.4 % decrease in hospital mortality and 37.7 % decrease in rates of cardio-pulmonary arrest outside of the PICU.^14^ Broadly, the literature indicates that implementation of a paediatric MET brings emergency personnel to deteriorating patients sooner, and this is associated with reduced in-hospital cardiac arrests and mortality, however these studies acknowledge the lack of robust evidence and the inherent variability of rapid response systems between hospitals.

A 2015 *meta*-analysis (mixed adult and paediatric) showed a reduction in mortality following RRT implementation, but noted that most of the included studies were observational before-and-after studies; there was significant heterogeneity; and insufficient studies for subgroup analysis based on study design.^21^

We chose to utilise an interrupted time series design with segmental regression analysis in our study. Interrupted time series methodology is increasingly being used for the evaluation of health interventions and is particularly suited to interventions introduced at a population level over a clearly defined time period and that target population-level health outcomes, such as mortality rates.^22^ This method of analysis helped to account for potential confounders and provided a clear picture of the profound impact of the intervention in the period immediately after implementation. The impact on hospital mortality was then sustained in the longer term. The counterfactual model suggests that, after 20 years, hospital mortality rate may have been three times higher than experienced, had the RRS not been implemented.

As one of the earliest paediatric hospitals to initiate a MET system (in 1994) and with the longest duration single-centre cohort of prospectively recorded paediatric MET events, this paper also establishes MET dose as an important factor that correlates with hospital mortality in the paediatric population.

MET Dose is well established in the adult literature as an important mediating factor in the effectiveness of an RRT. The first publication identifying a dose-related effect of MET implementation was a 2003 registry-based study wherein the incidence of fatal cardiopulmonary arrests in adults decreased from 4.3 to 2.2 per 1000 admissions when introduction of objective MET criteria caused a significant increase in MET dose.^15^ This association was also demonstrated in a prospective before-and-after study of MET implementation at The Austin Hospital in Melbourne, finding an inverse correlation between the number of MET calls and number of cardiac arrests per calendar year.^17^ The MERIT study, a cluster randomised control trial of 23 Australian hospitals where 12 implemented a MET system, found no significant reduction in the composite primary outcome of cardiac arrest, unexpected death or unplanned ICU admission in the 6 month period after MET implementation.^23^ However, in post hoc analysis of this data, it was identified that for every 10 % increase in the proportion of early MET calls there was a 2.0 per 10,000 admissions reduction in unexpected cardiac arrests and 0.94 per 10,000 admissions reduction in unexpected deaths.^18^

Our findings necessitate further examination of the association between increased MET Dose and patient-centred outcomes. The observed association between increasing MET Dose and decreasing in-hospital mortality is not unexpected. Rapid Response Systems are designed to recognise clinical deterioration early and bring critical care clinicians to the patient earlier to prevent reversible deterioration. Despite these benefits, it is well recognised that multiple obstacles prevent bedside clinicians activating the RRS when they should. Potential barriers include but are not limited to: level of clinical experience of clinicians involved in RRS activation; alterations to RRS activation criteria; reluctance of medical reviews; distribution of resources and inadequate staffing; and poor communication and documentation.^24^

Conversely, as systems remove these obstacles and encourage bedside clinicians to escalate concern earlier, the likelihood of wasting resources on unnecessary MET activations increases ie. ‘false positives’. Whilst it was not observed in the current data, it is likely that a plateau exists in the inversely proportional relationship between MET dose and in-hospital mortality. A mature RRS may even observe a decrease in MET dose once systems are so refined that clinicians detect and respond to deterioration much earlier, predicting all that is predictable and preventing almost everything that is preventable, so that METs become a very rare event.

## Limitations

There are several limitations to our study. As with any observational cohort study, internal validity is limited by the potential for confounding factors (other than RRS implementation) that may have influenced in-hospital mortality at RCH Brisbane during the study period. It is also possible that ancillary benefits of RRS implementation, such as improved education of nursing and junior medical staff on the identification of clinical deterioration is an important mediating factor of RRS effectiveness. The external validity is also limited as data was derived from a single tertiary paediatric hospital, and as such must be interpreted in concert with multi-centre data. We also acknowledge that that the period over which these data span ended over a decade ago hence the generalisability may have diminished with further changes in our ability to recognise and prevent deterioration.

## Conclusions

Utilising the data from one of the earliest and longest duration single-centre cohort of paediatric MET events, this study reaffirms the association between implementation of a paediatric RRS and decreased hospital mortality. The study also provides novel evidence of the impact of MET dose on patient outcome in the paediatric population. It is recommended that factors influencing the benefit of Rapid Response Systems in paediatric populations are further identified so that this life saving initiative can be optimised.

## CRediT authorship contribution statement

**Jason Acworth:** Writing – review & editing, Writing – original draft, Supervision, Project administration, Methodology, Data curation, Conceptualization. **Connor Ryan:** Writing – original draft, Visualization. **Elliott Acworth:** Writing – review & editing, Visualization, Methodology. **Syeda Farah Zahir:** Writing – review & editing, Visualization, Formal analysis.

## Funding

This research did not receive any specific grant from funding agencies in the public, commercial, or not-for-profit sectors.

## Declaration of competing interest

The authors declare that they have no known competing financial interests or personal relationships that could have appeared to influence the work reported in this paper.
